# Factors associated with nurses’ performance of oral assessments and dental referrals for hospital inpatients

**DOI:** 10.1186/s12903-020-1058-0

**Published:** 2020-03-12

**Authors:** Satoru Haresaku, Souhei Uchida, Hisae Aoki, Kazuyuki Akinaga, Rie Yoshida, Keiko Kubota, Toru Naito

**Affiliations:** 1 Department of Nursing, Fukuoka Nursing College, 2-15-1 Tamura, Sawara-ku, Fukuoka, 814-0193 Japan; 2 Section of Geriatric Dentistry, Department of General Dentistry, Fukuoka Dental College, 2-15-1 Tamura, Sawara-ku, Fukuoka, 814-0193 Japan

**Keywords:** Dental referral, Oral assessment, Nurse, Oral health care, Oral health service

## Abstract

**Background:**

Nurses’ oral assessment and dental referral performance for inpatients are important to provide appropriate oral care services in hospitals. The purpose of this study was to investigate the knowledge, attitudes, and performance of oral assessments and dental referrals for their inpatients among nurses and to identify factors associated with that performance to promote oral health care in hospitals.

**Methods:**

All nurses (*n* = 919) who worked at five hospitals in Japan were recruited as responders. A questionnaire regarding their performance of oral assessments and dental referrals was distributed to the subjects in each hospital. The data were collected from August 2018 to September 2018.

**Results:**

A total of 757 (82.4%) nurses (82 males and 675 females) responded to the questionnaire. With respect to each of the 8 oral assessment categories, 16.2–41.2% of the nurses performed oral assessments for more than 50% of their inpatients, and 20.3–29.9% had encouraged more than one inpatient to see a dentist within the previous 3 months. Significant differences were found by ward and hospital in their performance of oral assessments for inpatients. Additionally, their oral assessment performance, knowledge of the usage of oral assessment tools, wards, and hospitals were significantly associated with their dental referral performance.

**Conclusions:**

The performance of oral assessment and dental referral was not developed sufficiently in the hospitals. Thus, oral health professionals should support oral assessment education for nurses, including usage of assessment tools, to promote dental referral by nurses. These results may contribute to promotion of dental referral performance by nurses and provision of oral health care by oral health professionals for hospital inpatients.

## Background

Oral health care is effective for the prevention of aspiration pneumonia and ventilator-associated pneumonia (VAP) [1–3]. In addition, a previous study reported that professional oral health care reduced oral mucositis pain in patients treated with chemotherapy [4]. Therefore, collaborations between oral health professionals and other health care staff in hospitals are needed to provide appropriate oral health care to inpatients and thus prevent those conditions.

However, the performance of regular oral health checkups by dentists to detect oral problems in hospitals may be difficult, especially for hospitals without a dental department. A previous statistical survey in Japan reported that only 26.8% of 1952 hospitals had a dental department [5]. A previous study reported that the inability to perform regular oral health checkups for inpatients was a major barrier for the provision of appropriate dental care for these patients [6].

To resolve this problem, oral assessments and dental referrals performed by health care workers who usually provide medical care for inpatients, such as nurses, may play a more important role than oral health checkups by dentists. Nurses can be good providers of oral assessments and can serve as a bridge by referring inpatients with oral problems [7–13]. Therefore, it is suggested that the development of nurses’ oral assessment performance for their inpatients will provide opportunities to increase their dental referrals and the provision of oral health care for these inpatients.

Oral assessment tools have been developed for the performance of oral assessment by non-oral health professionals to identify the need for dental referrals [7–11]. Oral assessments by non-oral health care professionals, such as nurses, have been shown to be valid and reliable. However, a previous study of nurses’ performance of oral assessments at a hospital reported that only 51.7% of 143 nurses had performed oral assessments for elderly patients and that only one of them had used an oral assessment tool [12]. The results of this study indicated a low performance of oral assessments by nurses and limited use of the available tools. Although some studies are available regarding nurses’ oral assessment performance [7–13], few have focused on the use of oral assessment tools for dental referral by nurses [10]. In addition, no studies have investigated the association between nurses’ oral assessments and dental referrals and the factors associated with their performance.

The purpose of this study was to investigate the knowledge, attitudes, and performance of inpatients’ oral assessments and dental referrals by nurses and to identify factors associated with that performance to promote oral health care in hospitals.

## Methods

### Design and sample

This study was a cross-sectional survey of nurses who worked in five hospitals in Fukuoka prefecture, Japan, from August to September 2018. Fukuoka Prefecture is situated on the northern shore of the Japanese island Kyushu. A nursing school to which the author belonged had 28 collaborative hospitals for nursing research and nursing students’ education. Five general hospitals (A, B, C, D, and E) were selected because they had many wards. Hospital A was also selected because it was the only hospital with a dental department. Hospital A was a member of the National Hospital Organization and was located in the center of a mid-sized city. Hospital B was a city hospital and was located in the center of the prefectural capital. Hospitals C, D, and E were located in rural districts. Hospitals A, B, C, D, and E had 591, 204, 402, 182, and 181 beds, respectively. Nurses who worked in the severe mental and physical disabilities wards (160 hospital beds) in hospital C were excluded because of their special clinical situation. All the other nurses were enrolled in this survey. No power calculation was performed.

### Structured questionnaires

The questionnaire items were derived from a previously developed questionnaire and were used to study the practices, attitudes, and confidence of nursing professionals in performing oral assessments [12]. The validity and reliability of the questionnaires were checked in previous studies [12, 13]. Questions regarding nurses’ performance of dental referrals for their inpatients were added to the questionnaire. Prior to the study, the questionnaire was pilot-tested with 10 nurses who worked at a nursing school and had more than 5 years of experience working at a hospital and were not included as subjects in this study.

The collected socio-demographic information included gender, age group, term (years) of work experience as a nurse, and workplace. The workplaces were divided into the following four categories: a) the perioperative period ward, b) the general ward, c) the outpatient section, and d) other wards (including the dialysis unit, mental ward, tuberculosis ward, and infectious diseases ward). In Japan, a perioperative ward (perioperative management center) is established to improve the clinical outcomes of patients who have undergone surgery in many university or general hospitals [14–16]. It comprises multi-professionals such as surgeons, dedicated nurses, anesthesiologists, dentists, physiotherapists, pharmacists, and nutritionists to perform intensive cross-sectoral perioperative management [14]. Medical insurance for perioperative oral function management, which was introduced in 2012, is available for inpatients during the perioperative period at the perioperative ward when they receive oral function management, such as dental treatments and oral health care by oral health professionals. The hospital can obtain the reimbursement for dental referrals for the inpatients. Therefore, insurance may affect the performance of oral assessments and dental referrals for these inpatients. For this reason, the perioperative wards (including the chemotherapy and radiation therapy wards) were separated from the general wards.

The questions regarding oral assessment and dental referral consisted of the following 4 parts: knowledge of oral assessment tools (2 categories), attitudes regarding oral assessment and dental referral (2 categories), performance of oral assessment for their inpatients (8 categories), and performance of dental referral for their inpatients (8 categories).

To assess the nurses’ knowledge of the usage of oral assessment tools, they were asked whether they were aware of the usage of two representative oral assessment tools [the OHAT (oral health assessment tool) and the OAG (oral assessment guide)], which are mainly used by nurses in Japan [10–13].

To evaluate the nurses’ attitudes in relation to the performance of oral assessments and dental referrals, they were asked whether they felt that a) nurses should perform oral assessments for oral health care and b) nurses should perform dental referrals for their patients. A four-point scale was used for the attitude towards dental referral (“Strongly agree”, “Somewhat agree”, “Somewhat disagree”, or “Strongly disagree”).

To assess their performance of oral assessments for their inpatients, the nurses were asked the percentage of their inpatients for whom they performed oral assessments for each of the assessment categories. The assessment categories, which were defined based on the OHAT [9], were as follows: a) the lip (swelling, bleeding, and ulceration), b) the tongue and tongue coating, c) the gingiva and oral mucosa (swelling, bleeding, and ulceration), d) saliva (quality and quantity), e) the present teeth (decayed teeth or tooth fracture), f) removable dentures (broken area), g) oral cleanliness (food debris, calculus, and plaque), and h) oral pain (verbal and/or non-verbal signs of pain). The examination of the oral categories of the OHAT by non-oral professionals (nurses) was shown to be valid and reliable in a previous study [9]. The percentages were divided into three categories (“≤10%”, “11–49%”, or “≥50%”).

To evaluate their performance of dental referrals for their patients, the nurses were asked how many inpatients they had encouraged to see a dentist within the 3 previous months in each assessment category (lip, tongue and tongue coating, gingiva and oral mucosa, saliva, present teeth, removable dentures, oral cleanliness, and oral pain). The numbers of inpatients were divided into three categories (“0”, “1–5”, or “≥6”).

All procedures performed in studies involving human participants were approved by the Ethics Committee of Fukuoka Gakuen, Fukuoka, Japan (approval No. 415) and were in accordance with the Ethical Guidelines for Clinical Research (the Ministry of Health, Labour and Welfare, Tokyo, Japan, No. 415 of 2008) and the 1964 Declaration of Helsinki and its later amendments or comparable ethical standards. The purpose of the study was explained to all the participants during the delivery of the anonymous self-reported questionnaire. A returned questionnaire was considered to be indicative of consent to participate.

### Data procedure

The questionnaires were distributed to all nurses through nurse staff members at all wards in the five hospitals in August 2018. The front page of the questionnaire explained the aim of the project and the voluntary nature of the study. If the nurse agreed to participate, then he/she answered the questionnaire and returned it to the boxes in the wards. The boxes were collected by the staff from August to September 2018.

### Statistical analysis

A Chi-squared test was used to explore differences in knowledge of the usage of the assessment tools, attitudes towards oral assessment and dental referral, performance of oral assessments for their inpatients, and performance of dental referrals for their inpatients among wards and hospitals. A logistic regression analysis with backward elimination was used to identify factors associated with their dental referral performance. The dependent variable was “nurses’ performance of dental referrals for their patients within the previous 3 months” in each assessment category and was divided into 2 variables (nurses’ encouraging more than one inpatient to see a dentist in the previous 3 months = 1 and never = 0). The independent variables were gender, age group, term of work experience as a nurse, ward, hospital, knowledge and attitudes regarding oral assessment and dental referral, and oral assessment performance for each assessment category.

Missing data were excluded from the analysis. The data were analyzed at the 5% significance level. The statistical analyses were performed using the IBM SPSS Statistics software program (version 21.0; IBM Corporation, Armonk, NY, USA).

## Results

A total of 757 nurses participated in this study. The total response rate was 82.4%, with a range from 76.3 to 90.2%. The majority (89.2%) were female (Table 1). The mean age was 36.1 years (SD = 10.6). The majority of the participants were in the age group (51.7%), and the most common term of work experience as a nurse (58.1%) was “< 11 years”. The percentages of the workplace groups were 10.6% in the perioperative ward, 64.5% in the general ward, 6.2% in the outpatient section, and 18.8% in the other wards. Significant differences were found in all categories of the characteristics among hospitals (*P* < 0.001).

**Table 1:** Characteristics of the subjects according to hospitals

	A, *n* = 245	B, *n* = 185	C, *n* = 167	D, *n* = 83	E, *n* = 77	Total, *n* = 757	
	n (%)	n (%)	n (%)	n (%)	n (%)	n (%)	*P*-value*
Gender							
Male	12 (4.9)	16 (8.6)	41 (24.6)	7 (8.4)	6 (7.8)	82 (10.8)	0.000
Female	233 (95.1)	169 (91.4)	126 (75.4)	76 (91.6)	71 (92.2)	675 (89.2)	
Age group							
< 36 y	162 (66.1)	91 (49.2)	73 (43.7)	38 (45.8)	27 (35.1)	391 (51.7)	0.000
≥ 36 y	83 (33.9)	94 (50.8)	94 (56.3)	45 (54.2)	50 (64.9)	366 (48.3)	
Term of work experience as a nursing							
< 11 y	178 (72.7)	104 (56.2)	82 (49.1)	47 (56.6)	29 (37.7)	440 (58.1)	0.000
≥ 11 y	67 (27.3)	81 (43.8)	85 (50.9)	36 (43.4)	48 (62.3)	317 (41.9)	
Workplace							
Perioperative ward	36 (14.7)	40 (21.6)	4 (2.4)	0 (0.0)	0 (0.0)	80 (10.6)	0.000
General ward	157 (64.1)	90 (48.6)	133 (79.6)	71 (85.5)	37 (48.1)	488 (64.5)	
Outpatient section	0 (0.0)	31 (16.8)	0 (0.0)	0 (0.0)	16 (20.8)	47 (6.2)	
Other	52 (21.2)	24 (13.0)	30 (18.0)	12 (14.5)	24 (31.2)	142 (18.8)	

*Chi-squared test

Regarding the knowledge of oral assessment tools, 24.7% of nurses knew about usage of the OHAT, and 10.0% knew about usage of the OAG (Table 2). The highest and lowest percentages of knowledge were 72.3% in hospital D and 8.4% in hospital C for the OHAT and 14.3% in hospital A and 6.5% in hospital E for the OAG, respectively. Significant differences by hospital were found in knowledge of usage of the OHAT (*P* < 0.001).

**Table 2:** Nurses’ knowledge and attitudes regarding oral assessment and dental referral

	A, *n* = 245	B, *n* = 185	C, *n* = 167	D, *n* = 83	E, *n* = 77	Total, *n* = 757	
	n (%)	n (%)	n (%)	n (%)	n (%)	n (%)	*P*-value*
Do you know about the usage of oral assessment tools?							
1) Usage of the OHAT (Oral health assessment tool)							
Yes	27 (11.0%)	79 (42.7%)	14 (8.4%)	60 (72.3%)	7 (9.1%)	187 (24.7%)	0.000
2) Usage of the OAG (Oral assessment guide)							
Yes	35 (14.3%)	14 (7.6%)	12 (7.2%)	10 (12.0%)	5 (6.5%)	76 (10.0%)	0.059
Do you feel that nurses should perform oral assessment for oral health care?							
Agree**	238 (97.1%)	173 (93.5%)	158 (94.6%)	79 (95.2%)	68 (88.3%)	716 (94.6%)	0.049
Do you feel that nurses should perform dental referral for their patients?							
Agree**	229 (93.5%)	129 (69.7%)	141 (84.4%)	63 (75.9%)	55 (71.4%)	617 (81.5%)	0.000

*Chi-squared test

**Total percentages of nurses choosing “Strongly agree” and “Somewhat agree”

Regarding attitudes towards oral assessment and dental referral, most of the nurses (94.6%) indicated that nurses should perform oral assessments for oral health care, and 81.5% indicated that nurses should perform dental referrals for their inpatients. The highest and lowest percentages of their attitudes were 97.1% in hospital A and 88.3% in hospital E for oral assessment and 93.5% in hospital A and 69.7% in hospital B for dental referral, respectively. Significant differences by hospital were found in their attitudes (*P* < 0.05 for the attitude towards oral assessment and *P* < 0.001 for the attitude towards dental referral).

Regarding the performance of oral assessments, 17.2–39.8% of the nurses performed oral assessments for more than 50% of their inpatients, although 25.4–61.0% performed assessments for less than 10% of their inpatients (Table 3). Nurses in hospitals A and D were more likely to perform oral assessments than nurses in the other hospitals, and significant differences by hospital were found in all assessment categories (*P* < 0.001).

**Table 3:** Percentages of nurses’ oral assessment performance for their inpatients according to assessment categories

Assessment categories	Oral assessment performance*	A, *n* = 245	B, *n* = 185	C, *n* = 167	D, *n* = 83	E, *n* = 77	Total, *n* = 757	
		n (%)	n (%)	n (%)	n (%)	n (%)	n (%)	*P*-value**
Lip	≤10%	29 (12.1)	73 (39.5)	46 (27.9)	15 (18.5)	27 (35.1)	190 (25.4)	0.000
	11–49%	83 (34.6)	56 (30.3)	63 (38.2)	30 (37.0)	28 (36.4)	260 (34.8)	
	50%≥	128 (53.3)	56 (30.3)	56 (33.9)	36 (44.4)	22 (28.6)	298 (39.8)	
Tongue and tongue coating	≤10%	28 (11.7)	85 (45.9)	53 (32.3)	19 (23.5)	27 (35.1)	212 (28.4)	0.000
	11–49%	83 (34.6)	59 (31.9)	70 (42.7)	34 (42.0)	31 (40.3)	277 (37.1)	
	50%≥	129 (53.8)	41 (22.2)	41 (25.0)	28 (34.6)	19 (24.7)	258 (34.5)	
Gingiva and oral mucosa	≤10%	34 (14.2)	91 (49.2)	49 (30.1)	25 (30.9)	34 (44.2)	233 (31.2)	0.000
	11–49%	96 (40.0)	58 (31.4)	74 (45.4)	33 (40.7)	26 (33.8)	287 (38.5)	
	50%≥	110 (45.8)	36 (19.5)	40 (24.5)	23 (28.4)	17 (22.1)	226 (30.3)	
Saliva	≤10%	45 (18.8)	117 (63.2)	67 (41.1)	29 (35.8)	42 (54.5)	300 (40.3)	0.000
	11–49%	104 (43.5)	44 (23.8)	65 (39.9)	36 (44.4)	27 (35.1)	276 (37.0)	
	50%≥	90 (37.7)	24 (13.0)	31 (19.0)	16 (19.8)	8 (10.4)	169 (22.7)	
Present teeth	≤10%	53 (22.1)	106 (57.3)	57 (35.0)	26 (32.1)	32 (41.6)	274 (36.7)	0.000
	11–49%	101 (42.1)	47 (25.4)	73 (44.8)	34 (42.0)	29 (37.7)	284 (38.1)	
	50%≥	86 (35.8)	32 (17.3)	33 (20.2)	21 (25.9)	16 (20.8)	188 (25.2)	
Removable dentures	≤10%	64 (26.7)	93 (50.3)	69 (42.6)	18 (22.2)	38 (49.4)	282 (37.9)	0.000
	11–49%	82 (34.2)	53 (28.6)	60 (37.0)	33 (40.7)	25 (32.5)	253 (34.0)	
	50%≥	94 (39.2)	39 (21.1)	33 (20.4)	30 (37.0)	14 (18.2)	210 (28.2)	
Oral cleanliness	≤10%	38 (15.8)	99 (53.5)	51 (31.3)	19 (23.5)	34 (44.2)	241 (32.3)	0.000
	11–49%	95 (39.6)	53 (28.6)	66 (40.5)	36 (44.4)	28 (36.4)	278 (37.3)	
	50%≥	107 (44.6)	33 (17.8)	46 (28.2)	26 (32.1)	15 (19.5)	227 (30.4)	
Oral pain	≤10%	107 (44.6)	139 (75.1)	114 (69.9)	30 (37.0)	65 (84.4)	455 (61.0)	0.000
	11–49%	59 (24.6)	30 (16.2)	32 (19.6)	32 (39.5)	10 (13.0)	163 (21.8)	
	50%≥	74 (30.8)	16 (8.6)	17 (10.4)	19 (23.5)	2 (2.6)	128 (17.2)	

*Percentage of their inpatients they performed oral assessment

**Chi-squared test

The nurses’ oral assessment performance between the perioperative and general wards was compared for hospitals A and B because few or no nurses worked in the perioperative wards in hospitals C, D, and E (Table 4). The percentages of nurses who performed oral assessments for less than 10% of their patients in hospital A were 13.9–27.8% in the perioperative ward and 12.4–22.2% in the general ward. Conversely, the percentages in hospital B were 45.0–72.5% in the perioperative ward and 52.2–25.6% in the general ward. Although nurses in the perioperative ward were less likely to perform oral assessments than nurses in the general wards in both hospitals, significant differences in the performance were found between the perioperative and general wards only in hospital B (*P* < 0.05).

**Table 4:** Comparison of nurses’ oral assessment performance for their inpatients between perioperative and general ward

	A (Having a dental department)				B (Having no dental department)			
Assessment categories	Oral assessment performance*	Perioperative ward, *n* = 36	General ward, *n* = 157	*P*-value**	Oral assessment performance*	Perioperative ward, *n* = 40	General ward, *n* = 90	*P*-value**
Lip	≤10%	6 (16.7)	19 (12.4)	0.418	≤10%	18 (45.0)	23 (25.6)	0.027
	11–49%	16 (44.4)	56 (36.6)		11–49%	8 (20.0)	38 (42.2)	
	50%≥	14 (38.9)	78 (51.0)		50%≥	14 (35.0)	29 (32.2)	
Tongue and tongue coating	≤10%	5 (13.9)	19 (12.4)	0.955	≤10%	21 (52.5)	29 (32.2)	0.058
	11–49%	14 (38.9)	58 (37.9)		11–49%	9 (22.5)	37 (41.1)	
	50%≥	17 (47.2)	76 (49.7)		50%≥	10 (25.0)	24 (26.7)	
Gingiva and oral mucosa	≤10%	7 (19.4)	22 (14.4)	0.550	≤10%	24 (60.0)	30 (33.3)	0.017
	11–49%	17 (47.2)	66 (43.1)		11–49%	9 (22.5)	36 (40.0)	
	50%≥	12 (33.3)	65 (42.5)		50%≥	7 (17.5)	24 (26.7)	
Saliva	≤10%	8 (22.2)	30 (19.7)	0.759	≤10%	29 (72.5)	47 (52.2)	0.056
	11–49%	18 (50.0)	70 (46.1)		11–49%	5 (12.5)	28 (31.1)	
	50%≥	10 (27.8)	52 (34.2)		50%≥	6 (15.0)	15 (16.7)	
Present teeth	≤10%	10 (27.8)	34 (22.2)	0.455	≤10%	26 (65.0)	39 (43.3)	0.016
	11–49%	18 (50.0)	69 (45.1)		11–49%	5 (12.5)	33 (36.7)	
	50%≥	8 (22.2)	50 (32.7)		50%≥	9 (22.5)	18 (20.0)	
Removable dentures	≤10%	7 (19.4)	30 (19.6)	0.825	≤10%	19 (47.5)	35 (38.9)	0.112
	11–49%	16 (44.4)	60 (39.2)		11–49%	7 (17.5)	32 (35.6)	
	50%≥	13 (36.1)	63 (41.2)		50%≥	14 (35.0)	23 (25.6)	
Oral cleanliness	≤10%	6 (16.7)	24 (15.7)	0.497	≤10%	26 (65.0)	37 (41.1)	0.032
	11–49%	18 (50.0)	62 (40.5)		11–49%	7 (17.5)	33 (36.7)	
	50%≥	12 (33.3)	67 (43.8)		50%≥	7 (17.5)	20 (22.2)	

*Percentage of their inpatients they performed oral assessment

**Chi-squared test

Regarding dental referral performance, only 16.0–24.6% of nurses had encouraged more than one inpatient to see a dentist within the previous 3 months (Table 5). Nurses in hospitals A and D were more likely to perform dental referrals for their inpatients than nurses in the other hospitals, and significant differences by hospital were found in all assessment categories (*P* < 0.05).

**Table 5:** Number of inpatients encouraged to see a dentist by each nurse within 3 months according to assessment categories

Assessment categories	Number of inpatients	A, *n* = 245	B, *n* = 185	C, *n* = 167	D, *n* = 83	E, *n* = 77	Total, *n* = 757	
		n (%)	n (%)	n (%)	n (%)	n (%)	n (%)	*P*-value*
Lip	0	195 (79.6)	165 (89.2)	135 (81.3)	59 (71.1)	61 (79.2)	615 (81.3)	0.006
	1–5	33 (13.5)	19 (10.3)	25 (15.1)	19 (22.9)	14 (18.2)	110 (14.6)	
	6≥	17 (6.9)	1 (0.5)	6 (3.6)	5 (6.0)	2 (2.6)	31 (4.1)	
Tongue and tongue coating	0	193 (78.8)	171 (92.4)	138 (83.1)	63 (75.9)	65 (84.4)	630 (83.3)	0.009
	1–5	35 (14.3)	13 (7.0)	19 (11.4)	15 (18.1)	8 (10.4)	90 (11.9)	
	6≥	17 (6.9)	1 (0.5)	9 (5.4)	5 (6.0)	4 (5.2)	36 (4.8)	
Gingiva and oral mucosa	0	191 (78.0)	168 (90.8)	136 (81.9)	61 (73.5)	59 (76.6)	615 (81.3)	0.002
	1–5	36 (14.7)	16 (8.6)	22 (13.3)	17 (20.5)	16 (20.8)	107 (14.2)	
	6≥	18 (7.3)	1 (0.5)	8 (4.8)	5 (6.0)	2 (2.6)	34 (4.5)	
Saliva	0	202 (82.8)	168 (90.8)	137 (82.5)	62 (74.7)	65 (84.4)	634 (84.0)	0.028
	1–5	26 (10.7)	16 (8.6)	20 (12.0)	16 (19.3)	8 (10.4)	86 (11.4)	
	6≥	16 (6.6)	1 (0.5)	9 (5.4)	5 (6.0)	4 (5.2)	35 (4.6)	
Present teeth	0	174 (71.0)	163 (88.1)	121 (72.9)	53 (63.9)	59 (76.6)	570 (75.4)	0.000
	1–5	54 (22.0)	21 (11.4)	37 (22.3)	26 (31.3)	14 (18.2)	152 (20.1)	
	6≥	17 (6.9)	1 (0.5)	8 (4.8)	4 (4.8)	4 (5.2)	34 (4.5)	
Removable dentures	0	184 (75.1)	166 (89.7)	125 (75.3)	48 (57.8)	57 (74.0)	580 (76.7)	0.000
	1–5	45 (18.4)	17 (9.2)	33 (19.9)	31 (37.3)	17 (22.1)	143 (18.9)	
	6≥	16 (6.5)	2 (1.1)	8 (4.8)	4 (4.8)	3 (3.9)	33 (4.4)	
Oral cleanliness	0	198 (80.8)	166 (89.7)	127 (76.5)	58 (69.9)	66 (85.7)	615 (81.3)	0.002
	1–5	29 (11.8)	16 (8.6)	30 (18.1)	20 (24.1)	7 (9.1)	102 (13.5)	
	6≥	18 (7.3)	3 (1.6)	9 (5.4)	5 (6.0)	4 (5.2)	39 (5.2)	
Oral pain	0	189 (77.1)	171 (92.4)	127 (76.5)	58 (69.9)	55 (71.4)	600 (79.4)	0.000
	1–5	44 (18.0)	13 (7.0)	36 (21.7)	22 (26.5)	19 (24.7)	134 (17.7)	
	6≥	12 (4.9)	1 (0.5)	3 (1.8)	3 (3.6)	3 (3.9)	22 (2.9)	

*Chi-squared test

Table 6 shows factors associated with the nurses’ dental referral performance. Performance of oral assessments and employment in the perioperative ward were significantly associated with performance of dental referrals in all assessment categories except for oral pain (*P* < 0.05). Hospital B was a negative independent variable associated with performance in many assessment categories, including gingiva and oral mucosa, present teeth, removable dentures, oral cleanliness, and oral pain (*P* < 0.05). The nurses’ knowledge of usage of the OAG was significantly associated with the performance for tongue and tongue coating (*P* = 0.017), and their knowledge of usage of the OHAT was significantly associated with the performance for removable dentures (*P* = 0.023). Other variables, such as age group, sex, and hospital C, were also significantly associated with performance (*P* < 0.05).

**Table 6:** Factors associated with nurses’ performance of dental referral

Dependent variables	Independent variables	odds ratio (95% CI*)	*P*-value**
Lip	Performing assessment of lip (R^†^ = ≤10%)	5.68 (1.75–18.44)	0.004
	Perioperative ward (R^†^ = other wards)	0.18 (0.06–0.59)	0.004
Tongue and tongue coating	Performing assessment of tongue (R^†^ = ≤10%)	6.33 (1.51–26.54)	0.012
	knowledge of the usage of OAG (R^†^ = No)	1.98 (1.13–3.46)	0.017
	Perioperative ward (R^†^ = other wards)	0.46 (0.25–0.84)	0.012
Gingiva and oral mucosa	Performing assessment of gingiva and oral mucosa (R^†^ = ≤10%)	4.52 (1.60–12.73)	0.004
	Male (R^†^ = female)	1.79 (1.01–3.18)	0.046
	Perioperative ward (R^†^ = other wards)	0.18 (0.05–0.57)	0.004
	Hospital B (R^†^ = other hospitals)	0.44 (0.25–0.77)	0.004
	Hospital C (R^†^ = other hospitals)	0.61 (0.37–0.98)	0.043
Saliva	Performing assessment of saliva (R^†^ = ≤10%)	5.45 (2.18–13.67)	0.000
	Perioperative ward (R^†^ = other wards)	0.20 (0.06–0.66)	0.008
Present teeth	Performing oral assessment of teeth (R^†^ = ≤10%)	5.16 (2.20–12.1)	0.000
	Perioperative ward (R^†^ = other wards)	0.45 (0.22–0.95)	0.035
	Hospital B (R^†^ = other hospitals)	0.44 (0.27–0.73)	0.001
Removable dentures	Performing assessment of removable dentures (R^†^ = ≤10%)	4.60 (2.26–9.35)	0.000
	knowledge of the usage of OHAT (R^†^ = No)	1.63 (1.07–2.48)	0.023
	Perioperative ward (R^†^ = other wards)	0.44 (0.20–0.95)	0.038
	Hospital B (R^†^ = other hospitals)	0.29 (0.17–0.51)	0.000
Oral cleanliness	Performing assessment of oral cleanliness (R^†^ = ≤10%)	6.89 (2.12–22.36)	0.001
	Perioperative ward (R^†^ = other wards)	0.36 (0.14–0.92)	0.033
	Hospital B (R^†^ = other hospitals)	0.53 (0.31–0.92)	0.023
Oral pain	Age group (R^†^ = 36≥)	1.86 (1.06–3.27)	0.032
	Hospital B (R^†^ = other hospitals)	0.28 (0.15–0.50)	0.000

*: Confidence interval

**: Logistic regression analysis with backward elimination

^†^: Reference = 0

## Discussion

This report is the first to investigate factors associated with nurses’ performance of oral assessments and dental referrals for their inpatients. Significant differences were found by hospital in all categories of the characteristics in this study. In the logistic regression analysis, sex, age group, and ward were significantly associated with the nurses’ dental referral performance. Therefore, those distributions in hospitals may affect their dental referral performance.

Less than 30% of nurses in the hospitals had knowledge about the usage of oral assessment tools. A previous study reported that only one nurse used the assessment tool [12]. Therefore, the tools were not used sufficiently in hospitals in Japan. We suggest that oral health professionals should support education in the use of the tools. Approximately 20% of the nurses did not indicate that nurses should provide dental referrals for their patients, although most of them felt that nurses should perform oral assessments. They may have felt that dental referrals were not part of their duty as nurses. Professional oral health care is important for the prevention of aspiration pneumonia and VAP [1–3]. Therefore, oral health professionals should educate hospital staff about the importance of dental referrals for their patients.

With respect to each of the 8 oral assessment categories, only 16.2–41.2% of the nurses performed oral assessments for more than 50% of their inpatients. The previous study regarding nurses’ oral assessment performance in a hospital reported that 51.7% of nurses performed oral assessments for elderly patients [12]. Because their performance of oral assessment for inpatients was not limited to “elderly inpatients” in this study, the performance percentages might be lower than those in the previous study. In Japan, most adults ≥30 years of age experience dental caries, and at least 40% of dentate adults aged ≥40 years have periodontal disease [17]. In addition, tooth loss increases starting at approximately 40 years of age [18]. Therefore, nurses should perform oral assessments and dental referrals for not only elderly inpatients but also younger generations to prevent dental diseases and tooth loss. Nurses in hospitals A and D were more likely to perform oral assessments than those in any of the other hospitals. The highest percentage of knowledge of usage of the OAG was 14.3% in hospital A, and the highest percentage of knowledge of usage of the OHAT was 72.3% in hospital D. Therefore, knowledge may be associated with performance.

The nurses who worked in the perioperative ward were less likely to perform oral assessments than those in the general ward. No previous studies have compared nurses’ oral assessment performance among wards. The nurses in the perioperative ward seem to be charged with many acute patients in severe conditions. Therefore, they may prioritize other important physical assessments over oral assessments, although further studies are needed to investigate the reasons. No significant difference in the performance of oral assessments was found between the perioperative and general wards in the hospital with a dental department, although significant differences were found in the hospitals without a dental department. Those results indicated that the presence of a dental department in the hospital might decrease the difference in the performance of oral assessments between the perioperative and general wards. Medical insurance for perioperative oral function management is only applied for inpatients during the perioperative period. Some studies reported that oral treatment by dentists and oral health care through the collaboration of health care staff were promoted in hospitals with a dental department after the introduction of insurance [15, 16]. Therefore, insurance may have affected the performance of oral health care, including oral assessment, in hospitals with a dental department.

With respect to each of the 8 oral assessment categories, only 20.3–29.9% of the nurses had encouraged more than one inpatient to see a dentist within the previous 3 months. At least 40% of dentate adults aged ≥40 years have periodontal disease [17]. Periodontal disease has been associated with cardiovascular diseases and is considered a cardiovascular risk factor [19]. Oral periodontopathic bacteria can be aspirated into the lung to cause aspiration pneumonia [20]. Therefore, the performance seemed to be low and unsatisfactory, and it should be promoted in all hospitals to prevent not only dental diseases but also general diseases.

Nurses in hospitals A and D were more likely to perform dental referrals for their patients than nurses in the other hospitals. The results were similar to the results of the oral assessment performance. In addition, the oral assessment performance was independently associated with the dental referral performance in all assessment categories except for oral pain. Inpatients seem to have difficulty recognizing the early stages of oral diseases, such as periodontal disease and oral cancer, and requesting dental treatment for themselves, because fewer subjective symptoms are present in the early stage [21, 22]. Therefore, nurses’ performance of oral assessments has been suggested to be one of the most significant detectors of oral diseases in inpatients and a promoter of dental referrals, suggesting that the development of oral assessments in hospitals may promote dental referrals for inpatients and the provision of oral health care.

The perioperative ward was negatively associated with nurses’ dental referrals in all assessment categories except for oral pain. Some studies reported that professional oral health care reduced oral mucositis pain in patients treated with chemotherapy concurrent with radiotherapy and that the inter-professional approach was associated with a sustained reduction of ventilator-associated pneumonia [23, 24]. Therefore, nurses should perform oral assessments for their patients and encourage those who have dental problems to see a dentist in the ward to prevent such diseases.

Hospital B was located in the center of the prefectural capital, and many dental clinics were located in close proximity. However, hospital B was a negative independent variable associated with nurses’ dental referrals in many assessment categories. Further research is needed to investigate the reasons for their low performance of dental referrals and to promote the performance in that hospital.

Knowledge of usage of the OAG was significantly associated with the nurses’ dental referral performance for the tongue and tongue coating. The OAG was developed as an oral assessment tool for patients undergoing cancer chemotherapy [7]. Squamous cell carcinoma is the most common oral malignancy, and the tongue is the most frequent location [25]. Patients undergoing chemotherapy commonly attribute difficulties maintaining food intake to development of an altered taste [26]. In addition, the tongue coating is easily assessed, and the validity and reliability of the assessment using this tool are high [7, 8]. Therefore, knowledge of usage of the OAG may promote dental referrals.

Knowledge of usage of the OHAT was significantly associated with the nurses’ dental referral performance for removable dentures. The assessment for removable dentures and teeth in the OHAT are divided into two categories, although those are a same assessment category in the OAG [7, 9]. Practical guidelines for physicians describe denture hygiene and maintenance as important for promotion of oral health in frail older adults [27]. That being the case, knowledge of OHAT usage may contribute to the promotion of dental referrals for dentures and oral health for frail inpatients with dentures.

Oral assessments and dental referrals were not developed sufficiently in the hospitals. A study conducted in 177 nursing schools reported that the average time for oral health care education in the curriculum was only approximately 2 h, and more than 80% of the educators were nurses [28]. It is expected that the nurses in this study had hardly received oral assessment education by oral health professionals when they were nursing students. It may contribute to their low performance on oral assessments and dental referrals. Therefore, oral health care education, including education on referring patients for oral assessment by oral health professionals in nursing curricula, is needed to promote oral assessments and dental referrals among nurses.

Several limitations associated with this study warrant mentioning. Participation in the study was voluntary. Therefore, an element of self-selection was present, and the data were self-reported. Maintaining anonymity was paramount, but unfortunately, the response rates were strongly affected by self-motivation to participate. The achieved overall response rate of 82.4% was within the normally accepted range for surveys [29].

Additionally, Japan had 7378 hospitals in 2017, but only five hospitals in one prefecture were selected without random sampling for this study. Therefore, selection bias might have occurred in the results of this study. We investigated only the nurses’ characteristics, knowledge and attitudes regarding the performance of oral assessments and dental referrals as the factors of performance. Other factors, such as their educational or community background, may also be associated with this performance. Additionally, differences may exist in the medical systems and the manner in which oral health care is provided to inpatients in each country. However, hospitals without a dental departments and the nurses’ oral assessment and dental referral performance will contribute to the provision of oral health care worldwide. Therefore, the results in this study may contribute to countries where the performance is not developed.

## Conclusion

This report is the first to describe factors associated with nurses’ oral assessment and dental referral performance for their inpatients in Japan. A questionnaire survey regarding oral assessments and dental referrals was administered to 919 nurses who worked at five general hospitals in Japan. A total of 757 (82.4%) nurses responded to the questionnaire. It showed that only 16.2–41.2% of the nurses performed oral assessments for more than 50% of their inpatients, and 20.3–29.9% had encouraged more than one inpatient to see a dentist within the previous 3 months. Their oral assessment performance, knowledge of the usage of oral assessment tools, wards, and hospitals were significantly associated with dental referral performance. Thus, oral health professionals should support oral assessment education for nurses and nursing students, including usage of tools, to promote dental referrals and provision of oral health care for hospital inpatients.

## Data Availability

The datasets generated and/or analyzed during the current study are not publicly available as the ethics approval was granted on the basis that only researchers involved in the study could access the de-identified data. Raw data have been stored securely at Fukuoka Nursing College.

## Abbreviations

OAG: Oral assessment guide
OHAT: Oral health assessment tool
